# 

**Published:** 1909-11-20

**Authors:** 


### Gifts and Bequests.

Mr. Robert Francis Wilkins has left £250 to the Dartmouth Cottage Hospital.

Alderman John Hubert Schmitz, of Croydon, left £100 to the Croydon General Hospital.

Mr. David Russell left £500 to the Ballymena Cottage Hospital for the Endowment Fund.

Mrs. Sarah Ann Rhodes has left £500 each to the Keighley and District Hospital and the Bradford General Infirmary.

Mrs. Mary Louisa Toby left £300 to the Devon and Exeter Hospital; and £200 each to the Exeter Dispensary and the Eye Infirmary, Exeter.

Mr. Edward Lonsdale Beckwith, of Eastbourne, left and bequeathed £250 to the Leaf Homœopathic Hospital, and £100 to the Princess Alice Memorial Hospital, both of Eastbourne. He also left £100 to the London Hospital.

The Treasurers of the Middlesex Hospital Cancer Charity have received from Mr. and Mrs. Richard R. Hollins a further donation of £100 for the maintenance of the "Richard Hollins" Scholarship attached to the Cancer Research Laboratories.

The Lord Mayor and the Treasurer of St. Bartholomew's Hospital acknowledge grants made by the Worshipful Companies of Merchant Taylors and Skinners, £1,000 each; Goldsmiths and Mercers, £500 each; Ironmongers and Salters, £105 each, for the liquidation of the capital debt of the hospital.

Another effort is being made with the help of Messrs. Harrods to aid at Christmas the following hospitals for sick and poor children :—The Children's Hospital, Great Ormond Street; the Queen's Hospital for Children, Dalston; the Victoria Hospital for Children, Chelsea; the Royal Waterloo Hospital for Children.

Miss Malham, of 3 Jamaica Street, Edinburgh, left £50 to the local Royal Infirmary.

Mr. J. Sharpe, of Rothermay, has left £50 to the Aberdeen Royal Infirmary.

Mr. Joseph Griggs, of Loughborough, left £100 each to the Loughborough Hospital and Dispensary and the Charnwood Forest Convalescent Homes.

Sir Ernest Shackleton has sent £100 to the Dreadnought Hospital, Greenwich, for sick and injured seamen, from the proceeds of the *Nimrod* exhibition.

The Treasurers of the Middlesex Hospital Cancer Charity have received from the Salters' Company the annual grant of £100 for the maintenance of the Salters' Scholarship attached to the Cancer Research Laboratories.

Sir Thomas Smith, Bart., K.C.V.O., F.R.C.S. (Eng.), late Hon. Serjeant Surgeon to the King, who died on October 1, left estate valued at £101,245 gross and £99,766 net. He left £30,000, the silver inkstand presented by his Majesty the King, and the silver fruit basket and gold watch presented to him by his house surgeons, with his portrait by Collier, in trust to follow the baronetcy.

Cardiff Infirmary.—Mrs. C. Gordon Canning and Mrs. Augusta E. Curre, the daughters of the late Mrs. Crawshay Bailey, Park Place, Cardiff, have given one thousand guineas for the purpose of endowing a cot at the Infirmary in memory of their mother. The sum of one hundred guineas has been received from Mr. John Sankey, K.C.—the seventh donation of a like amount since 1903, making a total contribution from Mr. Sankey of £735. Messrs. P. and A. Campbell, Bristol, have sent £55, being part proceeds of the annual charity trip run by them in September.

### APPOINTMENTS.

DR. F. J. SMITH has been appointed a consulting physician to the Poplar Hospital.

MR. HENRY WADE, M.D., F.R.C.S. (Eng.), has been elected assistant surgeon and Dr. James Miller, D.Sc., M.D., assistant pathologist to the Edinburgh Royal Infirmary.

### APPOINTMENTS VACANT.

WEST SUFFOLK EDUCATION COMMITTEE.—Medical inspector of school children.

SEAMEN'S HOSPITAL SOCIETY, GREENWICH.—Senior house surgeon; house surgeon.

KING EDWARD VII. HOSPITAL, WINDSOR.—Hospital secretary.

### The Question Box.

**SPECIAL NOTICE.**—Questions of interest affecting the honorary medical staff and hospital administration in all departments are invited. When any point arises we hope our readers will formulate it in a question and so co-operate to make this column of real value and interest. In this way the experience of the best-managed hospitals should be made helpful to the whole hospital world. We shall welcome the contribution of both questions and answers.

**N.B.**—The publication of an answer in this column does not necessarily preclude the publication of subsequent answers to the same question, for a question may involve points which require to be threshed out and fully discussed. Answers, if published, will be paid for at scale rates.

### HOSPITAL FLOORS AND LINOLEUM.

Question 2. What are the objections to the use of linoleum, and what is the best floor for hospital use?

Mr. P. Michelli, C.M.G., Secretary of the Dreadnought Hospital, Greenwich, writes :—

I have been interested in the discussion that has been going on in The Hospital on the subject of ward floors, and as I have had experience of most kinds—hard wood, soft wood, terrazzo tiles, and seamless floors, most of these with and without linoleum—I venture to say a word on the subject.

Terrazzo is too costly, too cold, and always cracks.

Tiles too cold and too many cracks.

Were it not that we are told by the profession that there are injurious microbes in dirt I would plump for the wood floor. There is something so very institutional about a wood floor; but it seems to me that the nearest thing to perfection is found in the new seamless floor. Come and see those that have been laid at the Dreadnought some two years ago—clean, cheap, cheerful, not a crevice where the most enterprising microbe can lodge, gentle to the nurses' feet, and apparently indestructible.

Linoleum we have tried in every way, and except as a means of covering up dirt I can see no advantage in it. It is bad enough at the London, but the methods suggested in the diagrams shown in Mr. Milburn's article appear to be designed specially with a view to prevent the dirt being cleaned out.

Mr. J. A. Rudd, Secretary of the Coventry and Warwickshire Hospital, Coventry, writes :—

In reference to the correspondence now appearing in your paper regarding hospital floors, my committee have been considering for a long time the most suitable floor from all points of view to be laid down in the new outpatient department now in course of erection at this hospital; and after visiting other hospitals where homogeneous floors have been laid, in some cases for a number of years, including Terrazzo, etc., they have decided on laying one of the jointless floors now under discussion in your paper. I might state that the manufacturers have given a satisfactory guarantee as to its durability, etc.

Mr. W. Milburn, jun., writes :—

Mr. H. Wilson Young in his letter to you published in your issue of November 13 appears to have gained an erroneous impression of a statement which I made in your issue of November 6 respecting the use of jointless flooring in German hospital wards.

What I there stated was that many so-called jointless floors, largely employed in German hospital wards a number of years ago on their invention, had now been practically discarded owing to their unreliability.

I in no sense referred to the jointless flooring trade in general, the great development of which in Germany I was well aware of, and of which Mr. Young gives most interesting particulars; nor did I refer in any way to any of the modern makes of jointless floor, the excellent qualities of many of which I am acquainted with, although I have not personally seem them employed in the great modern hospitals of such German cities as Berlin, Hamburg, Frankfort, Dresden, Nuremberg, or Munich.

THE
### GRADUATES' MEDICAL SCHOOLS.

DIARY FOR THE WEEK, NOVEMBER 22 to 27.

### ST. JOHN'S HOSPITAL FOR DISEASES OF THE SKIN, Leicester Square, W.C.

Chesterfield Lectures.—At the Hospital on Thursday evenings at 6 p.m., each lecture followed by clinical demonstrations :—

**Nov. 25. Syphilis** as it modifies other eruptions of the Skin : Symptoms, Diagnosis, and Treatment.

### LONDON SCHOOL OF CLINICAL MEDICINE, Seamen's Hospital, Greenwich, S.E.

**At 4 p.m.—Nov. 22,** Mr. R. Lake, **The Examination of the Ear.**

**At 3.15 p.m.—Nov. 23,** Mr. Carless, **Tubercular Disease of the Kidney.**

**At 3.30 p.m.—Nov. 24,** Mr. Cargill, **The Common Syphilitic Affections of the Eye.**

### MEDICAL GRADUATES' COLLEGE AND POLYCLINIC, 22 Chenies Street, W.C.

**At 4 p.m.—Nov. 22,** Dr. Wilfred Fox, **Skin.**

**At 5.15 p.m.—Nov. 22,** Dr. F. J. McCann, **Lecture-Demonstration on the Methods of Suturing a Ruptured Perineum.**

**At 4 p.m.—Nov. 23,** Dr. Guthrie Rankin, **Medical.**

**At 5.15 p.m.—Nov. 23,** Dr. Theo. Hyslop, **Diagnosis of Insanity.**

**At 4 p.m.—Nov. 24,** Mr. Lockhart Mummery, **Surgical.**

**At 5.15 p.m.—Nov. 24,** Dr. Leonard Williams, **The Diagnosis of Thyroid Inadequacy.**

**At 4 p.m.—Nov. 25,** Sir Jonathan Hutchinson, **Surgical.**

**At 5.15 p.m.—Nov. 25,** Dr. Stanley Barnes (Birmingham), **Diseases of the Cerebral Arteries.**

**At 4 p.m.—Nov. 26,** Dr. Dan Mackenzie, **Ear, Nose, and Throat.**

### THE HOSPITAL FOR SICK CHILDREN, Great Ormond Street, W.C.

**At 4 p.m.—Nov. 25,** Mr. Kellock, **The Minor Surgery of Children.**

### NORTH-EAST LONDON POST-GRADUATE COLLEGE, Prince of Wales's General Hospital, Tottenham, N.

**At 4.30 p.m.—Nov. 23,** Dr. A. E. Giles, **The Influence of the Menopause in Gynæcology.**

### NATIONAL HOSPITAL FOR THE PARALYSED AND EPILEPTIC, Queen Sq., Bloomsbury, W.C.

**At 3 30 p.m.—Nov. 23,** Dr. E. F. Buzzard, **Traumatic Epilepsy and its Treatment.**

**Nov 26,** Dr. E. F. Buzzard, **Multiple Neuritis.**

### THE POST-GRADUATE COLLEGE, West London Hospital, Hammersmith, W.

**At 10 a.m.—Nov. 22** and **25,** Surgical Registrar, **Demonstration.**

**Nov. 26,** Medical Registrar, **Demonstration.**

**At 12 noon.—Nov. 25,** Dr. Bernstein, **Pathological Demonstration.**

**At 12.15 p.m.—Nov, 23,** Dr. Pritchard, **Practical Medicine.**

**Nov. 24** and **27,** Dr. Grainger Stewart, **Practical Medicine.**

**At 5 p.m.—Nov. 22,** Mr. Baldwin, **Practical Surgery—III.**

**Nov. 24,** Dr. Low, **Insects as Carriers of Disease in the Tropics.**

**Nov. 25,** Mr. Keetley, **Unreduced Dislocations.**

**Nov. 26,** Mr. Armour, **Head Injuries.**

**At 3 p.m.—Nov. 23** (at the L.C. Asylum, Claybury, Essex), Dr. Robert Jones, **Types of Mental Diseases.**

### ROYAL SOCIETY OF MEDICINE, 20 Hanover Square, W.

**At 8 p.m.—Nov. 22, Odontological Section :—**

Dr. J. Eyre and Mr. J. Lewin Payne : "Some observations on the bacteriology of pyorrhœa alveolaris, and the results of treatment by bacterial vaccines."

Mr. C. Schelling : (1) "A case of fracture of the mandible, set with a silver splint made by the casting process." (2) "Two cases of suppuration in the maxilla, in which the Röntgen rays materially helped the diagnosis."

Mr. Stanley Boyd : "A case of unilateral overgrowth of the lower jaw." (The patient will be present at 7.30.)

**At 5.30 p.m.—Nov. 23, Medical Section :—**

Dr. F. Craven Moore and Mr. R. L. Ferguson : "The *rôle* of fats in the treatment of functional disorders of the stomach."

**At 8.30 p.m.—Nov. 25, Neurological Section :—**

Sir William Gowers, F.R.S. : "Special sense discharges in organic disease."

**At 4.30 p.m.—Nov. 26, Section for the Study of Disease in Children :—**

Mr. K. Bremer : "The variability and relative frequency of the lesions in polio-encephalomyelitis."

Mr. P. Lockhart Mummery : "A case of strangulation of the small intestine by a band in a child aged 15 months."

**At 8.30 p.m.—Epidemiological Section :—**

Mr. H. G. Armstrong : "Nine epidemics of measles at a public school."

Dr. Robert Milne : "Scarlet fever—its home treatment."

